# Hypodermic Use of Carbolic Acid in Rheumatism

**Published:** 1876-01

**Authors:** 


Hypodermic Use of Carbolic Acid in Rheumatism.—Some practitioners in Germany have for several years used subcutaneous injections of carbolic acid in rheumatism with great benefit, especially in the articular form. A solution is employed in the proportion of one to three parts of acid to 100 of water. It is injected near the part affected. No ill consequences have been noticed. The anæsthetic effect is remarkable, continuing five or six hours; and though it is not permanent, yet the cure of the disease is often greatly facilitated by this treatment.—*Pacific Med. and Surg. Jour.*

